# The heat goes on: Simplifying the identification of energy hardship

**DOI:** 10.1016/j.heliyon.2023.e19087

**Published:** 2023-08-11

**Authors:** Luiza Brabo-Catala, Anca Cernic, Eva Collins, Barry Barton

**Affiliations:** a Waikato Management School, University of Waikato, Hamilton, New Zealand; b Independent Researcher, New Zealand; c Te Piringa Faculty of Law, University of Waikato, Hamilton, New Zealand

**Keywords:** Energy hardship, Energy poverty, Fuel poverty, New Zealand, Energy efficiency, Energy affordability

## Abstract

Energy hardship affects over 6% of households in New Zealand, defined as the inability to afford and obtain energy services. In late 2021, the Ministry of Business, Innovation and Employment proposed 26 indicators to identify energy hardship. However, this study aimed to explore the leading causes and consequences of energy hardship in the following year, including relevant variables not included in the proposed indicators. A survey of 1278 Kiwi respondents was conducted to understand their demographic and energy consumption patterns. Using 17 of the proposed indicators, the severity of energy hardship was measured and related to other important variables. Results showed that energy unaffordability, poor housing quality, and choosing between food expenses or energy bills were the main drivers of energy hardship. Consequences included feeling cold due to restricted energy consumption and accumulating energy debt. This study provides valuable insights to simplify the identification of households experiencing energy hardship and highlights the main areas of focus for policy development aimed at eradicating this problem.

## Highlights

• 1278 Kiwi households were surveyed to identify energy hardship.
• We used 17 energy hardship indicators proposed by the government.
• Energy hardship drivers: food expenses, energy prices, and housing quality.
• Energy hardship effects: being cold from self-rationing and accruing energy debt.

## 1 Introduction

The residential electricity demand has increased in New Zealand [1], as well as its costs [2]. While virtually all homes in the country are connected to the electricity grid [3], the South Island (where roughly a quarter of the population lives [4]) has some particularities compared to the North Island, including no access to the gas grid [5], colder average temperatures [6], lower-income households [7], and higher usage of biomass and coal for heating [1,8].

It is estimated that over a hundred thousand households in New Zealand are unable to have adequate access to energy, corresponding to more than 6% of all households [9,10]. The country has poor housing quality, mainly relating to insufficient thermal insulation [[11], [12], [13]], and a high number of excess winter deaths [14]. In 2019, The New Zealand government released a document entitled, *Electricity Price Review*, which contained recommendations for understanding and minimising energy hardship in the country [9].

Energy hardship includes the lack of both affordability of energy access and of sufficient modern energy infrastructure [9]. The former issue is the most relevant to New Zealand, and it relates to the concept of *fuel poverty* used in the United Kingdom and *energy poverty* used in the European Union.

Two years after the *Electricity Price Review*, the Ministry of Business, Innovation and Employment (MBIE) proposed a definition and indicators to identify energy hardship in the country [15]. After consultation with the general public and experts, the condition is considered to be one extremity of a spectrum (not a binary condition), with the opposite end being *energy wellbeing*, officially defined as “[w]hen individuals, households and whānau [family, extended family, or community living together] are able to obtain and afford adequate energy services to support their wellbeing in their home or kāinga [Māori settlement]” [16].

The twenty-six MBIE energy hardship indicators were developed by a multidisciplinary team, which also received external feedback, focus on the three main origins of this condition: income, housing quality, and energy costs [15], including objective and subjective measures. We wanted to analyse the patterns in households that present those indicators to prioritise them, leading to simpler detection methods.

What are the common causes and consequences of energy hardship in Aotearoa [New Zealand]? To find the answer, we collected responses from a total of 1278 respondents (one from a nationally representative survey and another survey with customers of a social retailer) to identify common trends in energy hardship. We identified what issues are pushing households into energy hardship, as well as the results of the condition. Our findings can be used to create interventions to minimise both the causes and consequences of energy hardship.

In the next section, we describe the methods used for carrying out a regional and a local survey and their data analyses, followed by our survey findings. Subsequently, we discuss our survey results and its implications regarding energy hardship policies, being summarised in the conclusion.

## 2 Methods

To understand the severity of energy hardship, survey questions were related to the indicators proposed by MBIE [15] (see Appendix A for survey questions and Appendix B for MBIE indicators). Each MBIE indicator used was considered to be equal to one point on the energy hardship point score (EHPS) of each respondent. The higher the score, the further the respondent is from energy wellbeing. Other questions relating to issues associated with energy hardship were based on additional literature [[17], [18], [19], [20], [21], [22], [23], [24]].

All survey questions were tested and analysed by professionals from 14 different organisations, including government agencies, energy companies, non-profits, and research companies. Those organisations provided feedback on the survey itself, its delivery, and potential rewards. Ethics approval was provided in May 2022.

The first survey was sent to 2736 clients of the low-cost electricity retailer OurPower (Waikato region, North Island), which was selected because it is the retail brand of WEL Networks – a community-owned distribution company [25]. WEL Energy Trust holds 100% of the shares of WEL Networks, investing millions of NZD annually on grants for community projects and energy efficiency in the Waikato region [25,26].

From June 20, 2022 to July 10, 2022, we obtained 983 responses. Of those, 773 of those considered valid responses (completed the survey). The survey was shared via email from OurPower to their clients, and the email highlighted the survey reward: five respondents would be selected by draw to win one month of free electricity. Respondents were asked if they were interested in potentially receiving rewards, and those that said that were interested also agreed to respond to a follow-up survey in December 2022.

The second survey contained virtually the same questions as the first one, except for details relating to being an OurPower client, rewards, and correcting wording for clarity. Another modification was adding the answer option: *Do not know or prefer not to answer* rather than letting respondents skip questions. The second survey was shared via email by the data company Dynata, to a database of potential respondents (at least eighteen years old and responsible for making decisions regarding energy) as a nationally representative sample. Sample quotas based on region and ethnicity were used to ensure the final valid sample is representative of the New Zealand population, with gender and age not being considered as the responses represent the whole household. From 7 to September 19, 2022, we obtained 559 responses, and 505 of those were considered valid responses.

**Fig. 1:**
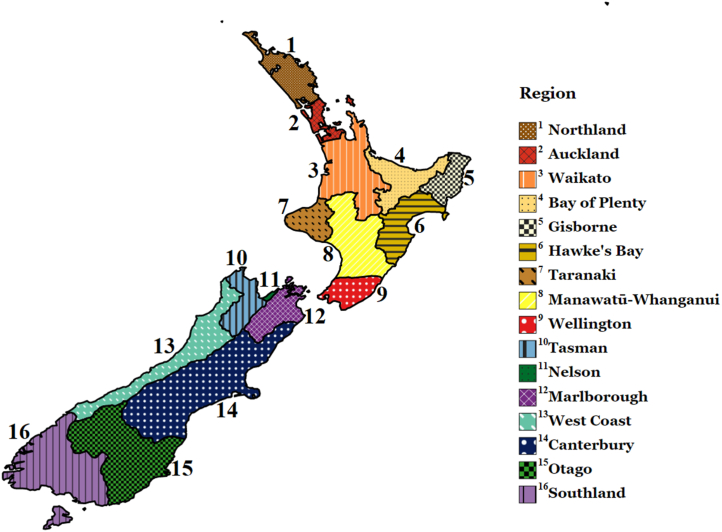
Map showing regions of New Zealand [27].

Ethics approval was granted by the Waikato Management School Human Research Ethics Committee, University of Waikato, prior to all answer collections (more details can be found in the Declarations section). Respondents were able to read about the purpose of the survey and were informed that they could skip any questions if they did not feel comfortable answering them. If they consented to participate, the survey would proceed. Otherwise, it would thank them and end. Besides OurPower, we were able to partner up with three local organisations to provide survey rewards: Habitat for Humanity in Northland and Auckland (non-governmental organisation), Northpower in Northland (community-owned electricity distributor), and Orion in Canterbury (community-owned electricity distributor). Orion was the only organisation located in the South Island (see Fig. 1 below for reference).

At the end of the second survey, if respondents with five or more EHPS in the mentioned regions, they were able to agree to obtain survey rewards. Rewards included energy advice, information on energy efficiency services and eligibility for subsidies, and energy-related items (e.g. education booklets, heaters, blankets, hot water bottles, draught stoppers, condensation squeegees, hygrometers, insulating curtains, and mould cleaning kits). Respondents who stated to be interested in potentially receiving rewards likewise agreed to respond to a follow-up survey in December 2022. Both surveys were conducted via the Qualtrics XM platform (Provo, Utah, USA) version June–December 2022.

Nine MBIE indicators were excluded due to access to electricity supply already being fully present in the OurPower survey (*no access to electricity supply*), overlap of indicators (*dampness and/or mould problems - major*), moving line nature (*proportion of AHC* [after housing costs] *household income spent on domestic energy costs twice the median or more (moving line)*, *proportion of BHC* [before housing costs] *household income spent on domestic energy costs twice the median or more (moving line)*, and *absolute domestic energy expenditure half the national median or less (moving line)*), ambiguous phrasing of question (*indoors always colder than would like in winter*), and respondent confusion when reporting their incomes (*proportion of AHC and BHC household income spent on domestic energy costs twice the median or more (moving and fixed lines*)) as well as whether they have an account with a financial institution and are connected to the electricity grid (*no access to financial institution account* and *no access to electricity supply*) [15].

Variables were classified as a cause of energy hardship according to O'Sullivan and Viggers [28] if they related to “housing quality, appliance efficiency, energy source and price, and occupant needs and income”, and the remaining variables were set as consequences. However, some issues can be both a cause and consequence and consequence of energy hardship, depending on the circumstances [29].

The statistical analysis to measure the strength of the relationship between the indicators used and other issues related to energy hardship were performed through Qualtrics. We analysed answer options separately, allowing the relationship between EHPS and variables to be used independently in further studies and policies, without being attached to all other variables.

When relating the EHPS with a group comparison of more than three or more options at a time, we used a more robust version of ANOVA, the one-way ANOVA test using Welch's F-test, as it can be used for both parametric and non-parametric data that does not have equal variance. For comparing EHPS with another group of unequal sample size and variance (comparing two options at a time, e.g. Māori selected and Māori unselected), two-tailed Welch's t-tests were used as they analyse the possibility for a substantial difference in means in either direction. However, if the distribution was not normal, sample size was too small, or if there were outliers, the *t*-test had to be ranked. When comparing EHPS to groups with data using exact numerical values, we used Pearson's r correlation for those with parametric data with high sample sizes and scalar or interval nature. Otherwise, Spearman's ρ ranked correlation was used for groups with non-parametric data, ordinal nature, and regardless of having a normal distribution or high sample size.

P-values and effect sizes of all analyses were used to determine which characteristics are the most relevant for determining if a Kiwi household is in energy hardship (p-value to see if there is a statistically relevant relationship between each variable and the EHPS, and the effect size to describe how strong the variable is). Additionally, a confidence interval (CI) of 95% was adopted for this study.

Due to the known inequality in material conditions, we hypothesised that some groups would be overrepresented in households in energy hardship: Māori, Pasifika, disabled people, tenants, residents of the South Island, and people with lower educational attainment [11,13,20,[30], [31], [32], [33]]. We had also expected to see that affected households to have inferior health outcomes [14,20,34,35].

## 3 Results

### 3.1 Energy hardship point scores

**Fig. 2:**
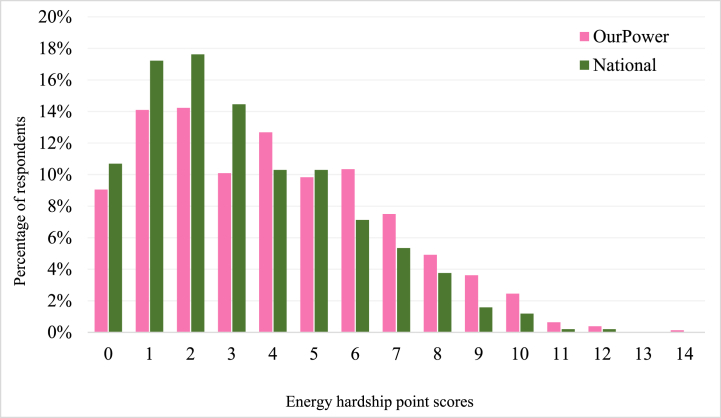
Distribution of energy hardship point scores across OurPower and national survey respondents.

Overall, the national survey respondents presented lower EHPS compared to OurPower respondents (Table 1 below). In both surveys the respondent distribution based on EHPS points was positively skewed (Fig. 2 below), with OurPower scores being more predominant after EHPS>5.

**Table 1:** Comparison of energy hardship point scores between OurPower and the national survey (SD = standard deviation).

Survey	Total (n)	Mode	Median	Average	CI	SD
OurPower	773	2.00	4.00	3.97	3.78 to 4.17	2.8
National	505	2.00	3.00	3.30	3.08 to 3.51	2.5

### 3.2 Describing households

#### 3.2.1 Location

The OurPower survey asked respondents for their postcode, being transformed into their equivalent cities/towns. One-way ANOVA test using Welch's F-test showed a statistically significant relationship between each city/town and the EHPS of respondents (P-value <0.00001), but the effect size is small (Cohen's f = 0.232). As most of these locations had small sample sizes, results at this level are unlikely to be representative.

Respondents of the national survey provided their region. However, one-way ANOVA test using Welch's F-test showed a P-value = 0.303 and a Cohen's f = 0.187, meaning there is not a statistically significant relationship between the region of respondents and EHPS.

#### 3.2.2 Household composition

Survey respondents provided the number of people living in their household in each of the age groups below. Even though the data was not ordinal, the distribution was not normal, so Spearman's ρ ranked correlation was the chosen analysis to relate the composition with the EHPS (Table 2 below).

**Table 2:** Relationship between number of people per age group and energy hardship point score (Spearman's ranked correlation).

Age group	Survey	Median number	Average number	CI of average number	SD	P-value	Effect size	CI of effect size
Below 5 years old	OurPower	0.0	0.4	0.36 to 0.47	0.7	0.000187	0.134	0.0641 to 0.203
	National	0.0	0.2	0.15 to 0.27	0.7	0.343	0.0423	−0.0451 to 0.129
5–14 years old	OurPower	0.0	0.8	0.72 to 0.90	1.3	0.0000567	0.144	0.0745 to 0.213
	National	0.0	0.3	0.24 to 0.35	0.7	0.0406	0.0912	0.00394 to 0.177
15–64 years old	OurPower	2.0	2.3	1.98 to 2.56	4.1	0.000309	0.129	0.0595 to 0.198
	National	2.00	1.8	1.66 to 1.97	1.7	0.000373	0.158	0.0715 to 0.242
65+ years old	OurPower	0.00	0.2	0.17 to 0.32	1.0	<0.00001	−0.162	−0.230 to −0.0924
	National	0.0	0.4	0.31 to 0.43	0.7	<0.00001	−0.217	−0.298 to −0.132

All the relationships are statistically significant, except for the *Below 5 years old* group in the national survey. All the effect sizes are small. The most significant effect sizes in both surveys were for the *65+ years old* group (ρ of −0.162 for OurPower and of −0.217 for national).

#### 3.2.3 Ethnicity

Survey respondents could select more than one ethnicity to describe all members of their households. Two-tailed Welch's t-tests were used to find the relationships between ethnicity and the EHPS (Table 3 below). Due to small sample sizes in some groups, not all analyses could be performed.

**Table 3:** Relationship between ethnicity and energy hardship point score (two-tailed Welch's *t*-test, with † being ranked *t*-test).

Ethnicity	Survey	Total (n)	Total (%)	Median EHPS	Average EHPS	CI of average EHPS	Average EHPS difference (unselected - selected)	CI of EHPS difference (unselected - selected)	P-value	Effect size
New Zealand European	OurPower	496	64.17%	3.00	3.30	3.08 to 3.53	1.88	1.48 to 2.28	<0.00001	0.711
	National	377	74.65%	3.00	3.14	2.90 to 3.39	0.599	0.0860 to 1.11	0.0223	0.243
Māori	OurPower	291	37.65%	5.00	5.17	4.84 to 5.51	−1.920	−2.32 to −1.52	<0.00001	0.727
	National	51	10.10%	4.00	4.25	3.47 to 5.04	−1.07	−1.88 to −0.251	0.0113	0.435
Pasifika	OurPower	73	9.44%	5.00	4.88	4.33 to 5.42	−0.994	−1.58 to −0.411	0.00104	0.357
	National	21	4.16%	4.00	4.14	3.05 to 5.23	−0.885	−1.99 to 0.224	0.112	0.358
Asian	OurPower	67	8.67%	3.00	3.39	2.80 to 3.98	0.645	0.0200 to 1.27	0.0432	0.231
	National	84	16.63%	3.00	3.19	2.68 to 3.70	0.125	−0.439 to 0.690	0.661	0.0507
Other	OurPower	61	7.89%	4.00	4.49	3.73 to 5.26	−0.56	−1.35 to 0.231	0.163	0.200
	National	49	9.70%	3.00	3.61	2.86 to 4.36	−0.351	−1.14 to 0.433	0.374	0.142
African	OurPower	11	1.42%	4.00	4.36	2.79 to 5.93	−0.393	−1.97 to 1.18	0.452†	0.197†
	National	3	0.59%	2.00	2.33	–	–	–	–	–
Latin American	OurPower	5	0.65%	4.00	4.00	3.12 to 4.88	−0.0235	−0.884 to 0.837	0.356†	0.133†
	National	0	0.00%	–	–	–	–	–	–	–
Middle Eastern	OurPower	2	0.26%	4.50	4.50	−1.85 to 10.85	–	–	–	–
	National	4	0.79%	4.00	3.75	2.95 to 4.55	−0.459	−1.20 to 0.280	0.0211†	0.425†
Do not know or prefer not to answer	National	1	0.20%	4.00	4.00	4.00 to 4.00	–	–	–	–

In both surveys, selecting Māori (as the ethnicity of at least one household member) had the highest effect sizes (Cohen's d of 0.727 for OurPower and 0.435 for the national survey). Not all ethnicities were statistically relevant when relating to EHPS.

#### 3.2.4 Education level

Survey respondents informed the highest level of education obtained by a household member, and a one-way ANOVA test using Welch's F-test measured the relationship between this variable and the EHPS. For OurPower respondents, the relationship was statistically significant (P-value<0.00001), with a moderate effect size (Cohen's f = 0.320). However, the relationship was insignificant for respondents to the national survey (P-value = 0.287 and Cohen's f = 0.109). Summarising, having lower education levels only translated to higher EHPS for OurPower respondents.

#### 3.2.5 General health

Survey respondents selected the option that best described the overall health of their household. Other than *Do not know or prefer not to answer*, the option *Good* was only available in the national survey. One-way ANOVA test using Welch's F-test was used to connect health and EHPS. Results showed P-values<0.00001 for both surveys, with a strong effect size for OurPower (Cohen's f = 0.455) and a moderate one for the national survey (Cohen's f = 0.338). This means that having poorer health outcomes is strongly connected to higher EHPS for OurPower respondents, whereas it is moderately connected to national survey respondents.

#### 3.2.6 Mental wellbeing

Survey respondents reported the how often they woken up feeling fresh and rested, and felt cheerful, in good spirits, calm, relaxed, active and vigorous, and that their daily lives has been filled with things that interest them (in the last two weeks). One-way ANOVA test on ranks linked their answers to their EHPS. OurPower and national survey responses presented statistically relevant relationships (P-values<0.00001) with large effect sizes (Cohen's f of 0.425 and 0.407, respectively).

#### 3.2.7 Disability/chronic illness

Survey respondents indicated if they were or had a disabled/chronically ill household member, being able to select more than one option according to the condition type. Two-tailed Welch's t-tests were run to establish the relationship between this variable and EHPS. The strongest relationship was found for those selecting *Intellectual* in the national survey (P-value = 0.0463 and Cohen's d = 0.760), but the sample size was small (n = 10). Not all conditions were considered statistically significant.

#### 3.2.8 Staying at home full-time

Survey respondents provided the number of household members staying at home full-time per age group. Pearson's r correlations and Spearman's ρ ranked correlations were performed to determine the strength of its relationships to the EHPS**.** For both surveys, the group *15 to 64 years old* presented the highest effect sizes (even though they are weak), with the OurPower ρ of 0.288 and the national one being 0.201. All the other groups from both surveys (except for *5 to 14 years old* in the OurPower survey) were not statistically relevant.

#### 3.2.9 Income

Survey respondents provided their household's annual income after tax, either by entering the number or selecting the brackets that best represented it. Then, all responses were compiled as income brackets for an one-way ANOVA test using Welch's F-test relating to the EHPS. Both surveys presented P-values<0.00001. The OurPower survey's effect size was large (Cohen's f = 0.476), whereas the national survey's was moderate (Cohen's f = 0.274).

#### 3.2.10 Affording necessities

Survey respondents of both surveys indicated whether the level their household's income was enough to afford everyday necessities (Table 4 below). Results linking them to the EHPS showed P-values<0.00001 and strong effect sizes, with Cohen's f = 0.697 for OurPower (one-way ANOVA test on ranks) and Cohen's f = 0.573 for the national survey (one-way ANOVA test using Welch's F-test).

**Table 4:** Relationship between household income being sufficient for affording necessities and energy hardship point score.

Income sufficiency for necessities	Survey	Total (n)	Total (%)	Median EHPS	Average EHPS	CI of average EHPS
More than enough money	OurPower	107	13.84%	1.00	1.68	1.33 to 2.03
	National	47	9.31%	1.00	1.43	0.99 to 1.86
Enough money	OurPower	207	26.78%	2.00	2.63	2.33 to 2.93
	National	160	31.68%	2.00	2.18	1.91 to 2.45
Just enough money	OurPower	277	35.83%	4.00	4.42	4.12 to 4.72
	National	204	40.40%	3.00	3.74	3.41 to 4.07
Not enough money	OurPower	181	23.42%	6.00	6.18	5.83 to 6.54
	National	88	17.43%	5.00	5.32	4.78 to 5.86
Do not know or prefer not to answer	National	6	1.19%	3.00	2.83	0.14 to 5.52

#### 3.2.11 Economising

Survey respondents were able to choose the action(s) their household performed to economise money. Two-tailed Welch's t-tests analysed the relationship between the options and the EHPS (Table 5 below).

**Table 5:** Relationship between actions performed to economise money and energy hardship point score (two-tailed Welch's *t*-test, with † being ranked *t*-test).

Action performed to economise money	Survey	Total (n)	Total (%)	Median EHPS	Average EHPS	CI of average EHPS	Average EHPS difference (unselected - selected)	CI of EHPS difference (unselected - selected)	P-value	Effect size
Spent less on hobbies or other special interests	OurPower	522	67.53%	4.00	4.48	4.24 to 4.71	−1.54	−1.94 to −1.14	<0.00001	0.568
	National	299	59.21%	3.00	3.77	3.49 to 4.05	−1.17	−1.60 to −0.744	<0.00001	0.486
Done without, or cut back on, trips to the shops or other local places	OurPower	476	61.58%	4.00	4.6	4.35 to 4.85	−1.62	−2.00 to −1.23	<0.00001†	0.624†
	National	259	51.29%	4.00	4.00	3.69 to 4.31	−1.45	−1.86 to −1.03	<0.00001	0.611
Delayed replacing, or repairing, broken or damaged appliances	OurPower	355	45.92%	5.00	5.06	4.77 to 5.35	−2.00	−2.37 to −1.62	<0.00001†	0.781†
	National	165	32.67%	4.00	4.55	4.17 to 4.92	−1.86	−2.30 to −1.41	<0.00001	0.801
Put up with feeling cold often	OurPower	295	38.16%	6.00	6.27	6.02 to 6.53	−3.71	−4.03 to −3.40	<0.00001	1.74
	National	153	30.30%	5.00	5.60	5.25 to 5.95	−3.31	−3.71 to −2.91	<0.00001	1.69
Gone without fresh fruit or vegetables	OurPower	272	35.19%	6.00	5.68	5.37 to 5.99	−2.63	−3.01 to −2.25	<0.00001	1.05
	National	150	29.70%	5.00	4.97	4.55 to 5.38	−2.38	−2.84 to −1.91	<0.00001	1.07
Postponed or put off visits to the doctor	OurPower	314	40.62%	5.50	5.46	5.16 to 5.76	−2.49	−2.86 to −2.12	<0.00001	0.991
	National	141	27.92%	4.00	4.65	4.27 to 5.03	−1.88	−2.33 to −1.43	<0.00001	0.809
None of those	OurPower	121	15.65%	1.00	1.65	1.36 to 1.94	2.76	2.40 to 3.12	<0.00001†	1.14†
	National	124	24.55%	1.00	1.60	1.33 to 1.88	2.24	1.87 to 2.61	<0.00001†	1.08†
Do not know or prefer not to answer	National	3	0.59%	4.00	3.67	−0.13 to 7.46	–	–	–	–

All options were statistically relevant, except for, *Do not know or prefer not to answer* and *Put up with feeling cold often* presented the highest effect sizes in both surveys (Cohen's d being 1.74 for OurPower and 1.69 for the national survey). And selecting *Put up with feeling cold often* increased the EHPS by one point.

#### 3.2.12 Housing situation

The OurPower survey asked the respondents about their housing situation (relating to homeownership/tenancy), being able to select more than one response, and two-tailed Welch's t-tests were used to connect them to the EHPS For the national survey, respondents were only able to select one answer that best represented their housing situation. Except for the *Other* group in the OurPower survey, all groups were statistically relevant with moderate effect sizes. The highest Cohen's d for OurPower was 0.788 for respondents who selected *Public housing*. One-way ANOVA test on ranks was used for the national survey's EHPS relationship analysis, resulting in a P-value<0.00001 and a medium effect size (Cohen's f = 0.379).

#### 3.2.13 Dwelling age

Respondents selected the decade which best represented when their dwelling was built. The relationships between them and the EHPS were performed using one-way ANOVA test using Welch's F-test. The P-values for the OurPower and national surveys were 0.0000607 and 0.0000297, respectively. The former's Cohen's f was 0.275, while the latter's was 0.363, meaning that the effect sizes were moderate.

#### 3.2.14 Dwelling amenities

Survey respondents selected which amenities were present in their dwelling, and two-tailed Welch's t-tests were used to measure the strength of the link between them and the EHPS (Table 6 below).

**Table 6:** Relationship between dwelling amenities or upgrades and energy hardship point score (two-tailed Welch's *t*-test, with † being ranked *t*-test).

Dwelling amenities	Survey	Total (n)	Total (%)	Median EHPS	Average EHPS	CI of average EHPS	Average EHPS difference (unselected - selected)	CI of EHPS difference (unselected - selected)	P-value	Effect size
Electricity grid connection	OurPower	675	87.32%	3.00	3.75	3.54 to 3.96	1.8	1.24 to 2.36	<0.00001	0.657
	National	432	85.54%	3.00	3.12	2.89 to 3.35	1.19	0.563 to 1.82	0.000289	0.489
Gas grid connection	OurPower	225	29.11%	3.00	3.15	2.82 to 3.47	1.17	0.766 to 1.57	<0.00001	0.424
	National	84	16.63%	2.00	2.87	2.38 to 3.36	0.511	−0.0367 to 1.06	0.0672	0.207
Drinkable tap water	OurPower	693	89.65%	3.00	3.65	3.45 to 3.84	3.19	2.55 to 3.83	<0.00001	1.21
	National	454	89.90%	3.00	3.11	2.88 to 3.33	1.85	1.18 to 2.53	<0.00001	0.768
Cooking facility with kitchen sink	OurPower	731	94.57%	4.00	3.82	3.62 to 4.02	2.91	1.98 to 3.85	<0.00001	1.07
	National	474	93.86%	3.00	3.15	2.93 to 3.37	2.40	1.60 to 3.20	<0.00001	0.997
Bathroom with toilet and shower/bath	OurPower	735	95.08%	4.00	3.81	3.61 to 4.00	3.46	2.56 to 4.36	<0.00001	1.28
	National	476	94.26%	3.00	3.14	2.92 to 3.36	2.72	1.88 to 3.57	<0.00001	1.140
Device with internet access	OurPower	673	87.06%	3.00	3.54	3.35 to 3.74	3.36	2.80 to 3.91	<0.00001	1.310
	National	471	93.27%	3.00	3.10	2.88 to 3.32	2.90	2.28 to 3.53	<0.00001	1.23
None of those	OurPower	11	1.42%	9.00	8.18	5.98 to 10.39	−4.27	−6.48 to −2.06	0.000103†	1.19†
	National	3	0.59%	5.00	5.33	−0.92 to 11.58	–	–	–	–
Do not know or prefer not to answer	National	8	1.58%	5.00	5.38	4.29 to 6.46	−2.11	−3.21 to −1.02	0.0000438†	0.983†

In the national survey, *Gas grid connection* was statistically insignificant. *Device with internet access* presented the highest effect sizes (Cohen's d of 1.310 for OurPower and 1.23 for the national survey). Selecting *None of those* counted as a point towards the EHPS. Otherwise, not selecting *Drinkable tap water*, *Cooking facility with kitchen sink*, *Bathroom with toilet and shower/bath*, or *Device with internet access* added one point each.

### 3.3 Describing energy usage

#### 3.3.1 Winter fuel

##### 3.3.1.1 Fuel type

Respondents of both surveys selected the type of fuel(s) used during winter months. Two-tailed Welch's t-tests were used to measure the relationship between the options and the EHPS Most answers were statistically insignificant. However, *Firewood, charcoal, or pellet* in the OurPower survey presented the highest effect size of those with a P-value<0.05, with a small Cohen's d of 0.290.

##### 3.3.1.2 Monthly fuel expense

Survey respondents typed their monthly winter fuel expenses per fuel type, and Pearson's r correlation and Spearman's ρ ranked correlation tests were performed to associate them with the EHPS**.** *Firewood, charcoal, or pellet* for OurPower respondents had the highest effect size (ρ = 0.294), being considered moderate. Many groups were statistically insignificant.

#### 3.3.2 Non-winter fuel

##### 3.3.2.1 Fuel type

Survey respondents selected the type of fuel(s) used during non-winter months. The relationship between the options and the EHPS was analysed using two-tailed Welch's t-tests. Many options could not be analysed due to their sample sizes. Of those that were statistically significant, after *Do not know or prefer not to answer*, the option *Electricity* in the national survey presented the strongest effect size (a medium Cohen's d of 0.643).

##### 3.3.2.2 Monthly fuel expense

Respondents of both surveys reported their monthly fuel expenses per fuel type (non-winter), and Pearson's r correlation was used to determine the strength of the relationship with the EHPS. Some options had small sample sizes and no analysis could be performed. *Electricity* for the national survey respondents had the only P-value<0.05, however, its effect size was negligible.

#### 3.3.3 Summer thermal comfort

Respondents were asked how often they felt their dwelling was too hot during the summer of 2021/2022. One-way ANOVA test using Welch's F-test was performed, and both surveys presented statistically significant relationship between the answers and the EHPS (OurPower's P-value<0.00001 and national's P-value = 0.00259). However, both had small and nearly identical effect sizes (OurPower's Cohen's f 0.177 and national's Cohen's f = 0.176).

#### 3.3.4 Winter thermal comfort

Respondents answered if they felt their home was always adequately warm during winter, as well as the reason(s) why if they selected no. Two-tailed Welch's t-tests analysed the relationships between the answers and the EHPS (Table 7 below).

**Table 7:** Relationship between winter thermal comfort and energy hardship point score (two-tailed Welch's t-tests, with † being ranked *t*-test).

Home adequately warm during winter	Survey	Total (n)	Total (%)	Median EHPS	Average EHPS	CI of average EHPS	Average EHPS difference (unselected - selected)	CI of EHPS difference (unselected - selected)	P-value	Effect size
Yes	OurPower	348	45.02%	2.00	1.89	1.72 to 2.05	3.81	3.53 to 4.09	<0.00001	1.84
	National	296	58.61%	2.00	1.92	1.73 to 2.11	3.33	2.98 to 3.67	<0.00001	1.79
No, due to financial reasons	OurPower	267	34.54%	6.00	6.23	5.96 to 6.50	−3.45	−3.79 to −3.11	<0.00001	1.52
	National	126	24.95%	6.00	5.79	5.42 to 6.15	−3.32	−3.73 to −2.90	<0.00001†	1.60†
No, due to energy inefficiency	OurPower	193	24.97%	6.00	5.91	5.55 to 6.26	−2.58	−2.99 to −2.17	<0.00001	1.00
	National	105	20.79%	5.00	5.19	4.75 to 5.63	−2.39	−2.89 to −1.90	<0.00001	1.05
No, due to another reason	OurPower	60	7.76%	5.00	5.03	4.28 to 5.79	−1.15	−1.93 to −0.371	0.0044	0.412
	National	17	3.37%	5.00	4.94	3.91 to 5.98	−1.70	−2.76 to −0.649	0.00326	0.693
Do not know or prefer not to answer	National	5	0.99%	4.00	4.20	2.36 to 6.04	−0.914	−2.74 to 0.914	0.125†	0.542†

Selecting *No (financial reasons)* counted as one point towards the EHPS. Selecting either *No (energy inefficiency)* or *No (another reason)* also added one point to the EHPS. In both surveys, selecting, selecting *Yes* had the largest effect sizes, with OurPower's Cohen's d being 1.84 and 1.79 for the national one.

#### 3.3.5 Heater type

Survey respondents selected the heater type(s) they own. Two-tailed Welch's t-tests were used to link the options with the EHPS (Table 8 below).

**Table 8:** Relationship between heater types and energy hardship point score (two-tailed Welch's *t*-test, with † being ranked *t*-test).

Heater type owned	Survey	Total (n)	Total (%)	Median EHPS	Average EHPS	CI of average EHPS	Average EHPS difference (unselected - selected)	CI of EHPS difference (unselected - selected)	P-value	Effect size
Heat pump	OurPower	620	80.21%	3.00	3.71	3.50 to 3.92	1.35	0.809 to 1.89	<0.00001	0.491
	National	338	66.93%	2.00	2.87	2.63 to 3.12	1.28	0.810 to 1.74	<0.00001	0.532
Electric heater	OurPower	384	49.68%	4.00	4.15	3.87 to 4.43	−0.347	−0.743 to 0.0486	0.0855	0.124
	National	258	51.09%	3.00	3.36	3.07 to 3.66	−0.142	−0.576 to 0.292	0.522	0.0572
Fixed gas heater	OurPower	69	8.93%	3.00	3.45	2.83 to 4.07	0.576	−0.0729 to 1.23	0.0812	0.206
	National	33	6.53%	3.00	3.36	2.50 to 4.23	−0.0734	−0.967 to 0.821	0.869	0.0296
Portable gas heater	OurPower	43	5.56%	7.00	6.79	5.92 to 7.66	−2.98	−3.87 to −2.10	<0.00001	1.100
	National	28	5.54%	4.00	4.36	3.44 to 5.27	−1.12	−2.06 to −0.185	0.0205	0.457
Wood burner	OurPower	118	15.27%	3.00	3.26	2.73 to 3.80	0.84	0.264 to 1.42	0.00453	0.301
	National	110	21.78%	3.00	3.49	2.97 to 4.01	−0.250	−0.823 to 0.323	0.389	0.101
Pellet fire	OurPower	1	0.13%	6.00	6.00	–	–	–	–	–
	National	4	0.79%	2.00	2.75	−0.78 to 6.28	0.549	−2.96 to 4.06	0.705†	0.203†
Coal burner	OurPower	4	0.52%	6.00	5.75	1.57 to 9.93	−1.79	−5.95 to 2.38	0.217†	0.673†
	National	2	0.40%	6.50	6.50	0.15 to 12.85	–	–	–	–
Other	OurPower	48	6.21%	3.00	3.06	2.33 to 3.79	0.972	0.216 to 1.73	0.0127	0.348
	National	21	4.16%	2.00	2.95	1.71 to 4.19	0.358	−0.901 to 1.62	0.561	0.144
None	OurPower	9	1.16%	7.00	6.89	4.91 to 8.87	−2.95	−4.93 to −0.969	0.00293†	0.987†
	National	14	2.77%	5.00	6.14	4.83 to 7.46	−2.93	−4.26 to −1.60	<0.00001†	1.11†
Do not know or prefer not to answer	National	2	0.40%	3.50	3.50	−2.85 to 9.85	–	–	–	–

Some options were not statistically relevant. Due to their small sizes, not all groups were analysed. *None* presented very strong effect sizes for the OurPower and national surveys (Cohen's d of 0.987 and 1.11, respectively). *Portable gas* had a high effect size for OurPower and a small one for the national survey (Cohen's d of 1.10 and 0.457, respectively). Selecting *Portable gas* option increased the EHPS by one point for being an indicator. If *None* was selected, that also increased their EHPS by one.

#### 3.3.6 Heated rooms

Survey respondents first selected which rooms they have in their dwellings, followed by selecting which rooms are heated during winter. The relationship between owned rooms that are heated and the EHPS was determined by two-tailed Welch's t-tests (Table 9 below).

**Table 9:** Relationship between owned rooms that are heated and energy hardship point score (two-tailed Welch's *t*-test, with † being ranked *t*-test).

Owned rooms that are heated	Survey	Total (n)	Total (%)	Median EHPS	Average EHPS	CI of average EHPS	Average EHPS difference (unselected - selected)	CI of EHPS difference (unselected - selected)	P-value	Effect size
Living room	OurPower	720	93.14%	3.00	3.78	3.58 to 3.97	2.94	2.12 to 3.76	<0.00001	1.09
	National	439	86.93%	2.00	2.98	2.77 to 3.20	2.24	1.43 to 3.05	<0.00001	0.954
Main bedroom	OurPower	385	49.81%	2.00	2.69	2.45 to 2.93	2.52	2.16 to 2.87	<0.00001	1.01
	National	251	49.70%	2.00	2.37	2.12 to 2.63	1.71	1.31 to 2.12	<0.00001	0.753
Children's bedroom	OurPower	334	43.21%	3.00	3.43	3.14 to 3.71	0.91	0.514 to 1.30	<0.00001	0.329
	National	126	24.95%	2.00	2.78	2.37 to 3.19	0.690	0.194 to 1.19	0.00657	0.284
Kitchen	OurPower	334	43.21%	2.00	2.93	2.66 to 3.21	1.790	1.41 to 2.16	<0.00001	0.675
	National	184	36.44%	2.00	2.40	2.08 to 2.72	1.31	0.887 to 1.73	<0.00001†	0.596†
Other bedroom	OurPower	123	15.91%	2.00	2.72	2.26 to 3.19	1.45	0.935 to 1.96	<0.00001	0.528
	National	73	14.46%	1.00	1.95	1.40 to 2.49	1.47	0.875 to 2.06	<0.00001	0.623
Bathroom	OurPower	108	13.97%	1.00	2.21	1.78 to 2.65	2.01	1.53 to 2.49	<0.00001†	0.798†
	National	82	16.24%	1.00	1.88	1.48 to 2.28	1.60	1.13 to 2.06	<0.00001	0.677
Office	OurPower	71	9.18%	1.00	1.96	1.45 to 2.46	2.18	1.64 to 2.73	<0.00001	0.803
	National	40	7.92%	1.00	1.83	1.23 to 2.42	1.50	0.854 to 2.14	0.0000206	0.628
Other room	OurPower	48	6.21%	2.00	2.29	1.58 to 3.00	1.76	1.02 to 2.49	<0.00001†	0.695†
	National	31	6.14%	1.00	1.68	0.85 to 2.51	1.68	0.819 to 2.54	0.000343	0.695
None	OurPower	18	2.33%	8.00	8.17	6.99 to 9.34	−4.33	−5.52 to −3.15	<0.00001	1.60
	National	17	3.37%	7.00	6.88	5.46 to 8.30	−3.80	−5.23 to −2.37	0.0000325	1.63
Do not know or prefer not to answer	National	5	0.99%	5.00	5.20	3.58 to 6.82	−2.01	−3.61 to −0.402	0.00394†	0.962†

*None* had the highest effect sizes for the OurPower and national surveys (Cohen's d of 1.60 and 1.63, respectively). Selecting *None* for heated rooms counted as a point towards the EHPS if the respondent did not answer *None* previously in the question about owning a heater. If the participant did not select heating *Living room*, *Main bedroom*, or *Children's bedroom* while having them, each of those unheated rooms added one point to the EHPS.

#### 3.3.7 Support equipment requiring energy

Respondents were asked about the frequency their household made use of medical, assistance, or mobility equipment at home which required energy consumption in the previous twelve months. The relationship between their answers and the EHPS was done via one-way ANOVA test using Welch's F-test. The national survey presented a high P-value of 0.0865 with a Cohen's f of 0.15, not being statistically significant. Yet, the OurPower survey had a P-value of 0.0000468 with a Cohen's f of 0.223, meaning the effect size was weak.

#### 3.3.8 Energy financial assistance

Respondents answered if they received any type of financial assistance towards energy in the past twelve months and their sources. Two-tailed Welch's t-tests were used to link the options and the EHPS (Table 10 below).

**Table 10:** Relationship between receiving financial assistance and energy hardship point score (two-tailed Welch's *t*-test, with † being ranked *t*-test).

Source of financial energy assistance	Survey	Total (n)	Total (%)	Median EHPS	Average EHPS	CI of average EHPS	Average EHPS difference (unselected - selected)	CI of EHPS difference (unselected - selected)	P-value	Effect size
Government	OurPower	228	29.50%	5.00	4.88	4.51 to 5.25	−1.28	−1.71 to −0.848	<0.00001	0.468
	National	148	29.31%	3.00	3.20	2.79 to 3.62	0.131	−0.354 to 0.615	0.596	0.0528
Family/friends gave or lent money	OurPower	65	8.41%	7.00	6.40	5.81 to 6.99	−2.65	−3.27 to −2.03	<0.00001	0.98
	National	16	3.17%	6.50	6.00	4.97 to 7.03	−2.79	−3.84 to −1.75	0.0000322	1.15
Took out a loan	OurPower	19	2.46%	6.00	6.16	4.96 to 7.35	−2.24	−3.45 to −1.03	0.001	0.805
	National	2	0.40%	6.50	6.50	−25.27 to 38.27	–	–	–	–
Energy provider	OurPower	42	5.43%	5.00	4.31	3.36 to 5.26	−0.355	−1.32 to 0.615	0.465	0.127
	National	2	0.40%	4.00	4.00	−8.71 to 16.71	–	–	–	–
Other	OurPower	28	3.62%	3.00	4.07	3.09 to 5.05	−0.101	−1.10 to 0.896	0.837	0.036
	National	1	0.20%	7.00	7.00	7.00 to 7.00	–	–	–	–
No assistance received	OurPower	478	61.84%	3.00	3.45	3.21 to 3.69	1.37	0.970 to 1.78	<0.00001	0.505
	National	339	67.13%	3.00	3.21	2.95 to 3.47	0.251	−0.223 to 0.726	0.298	0.102
Do not know or prefer not to answer	National	5	0.99%	4.00	3.60	2.18 to 5.02	−0.308	−1.71 to 1.09	0.243†	0.320†

Some sample sizes were too small, so analyses were performed for them. Not all options in both surveys presented P-values low enough to be statistically relevant. The highest effect sizes in the OurPower and national surveys were for the OurPower and national surveys were for *Family/friends gave or lent money* (Cohen's d of 0.98 and 1.15, respectively).

#### 3.3.9 Choosing between energy or other expenses

Survey respondents answered if they had to choose between paying energy bills or other expenses. Two-tailed Welch's t-tests were run to compare the relationship between each expense listed and the EHPS (Table 11 below).

**Table 11:** Relationship between paying energy bills or other expenses and energy hardship point score (two-tailed Welch's *t*-test, with † being ranked *t*-test).

Expense	Survey	Total (n)	Total (%)	Median EHPS	Average EHPS	CI of average EHPS	Average EHPS difference (unselected - selected)	CI of EHPS difference (unselected - selected)	P-value	Effect size
Hobbies/leisure	OurPower	208	26.91%	5.00	4.89	4.54 to 5.24	−1.22	−1.64 to −0.790	<0.00001	0.441
	National	95	18.81%	5.00	4.80	4.34 to 5.26	−1.85	−2.37 to −1.34	<0.00001	0.783
Clothing	OurPower	239	30.92%	6.00	5.56	5.22 to 5.89	−2.27	−2.67 to −1.86	<0.00001	0.871
	National	84	16.63%	6.00	5.36	4.85 to 5.86	−2.47	−3.02 to −1.93	<0.00001	1.08
Food	OurPower	230	29.75%	6.00	6.25	5.92 to 6.58	−3.23	−3.61 to −2.85	<0.00001	1.36
	National	81	16.04%	6.00	5.95	5.40 to 6.50	−3.16	−3.74 to −2.58	<0.00001	1.45
Transportation expenses	OurPower	230	29.75%	6.00	5.73	5.38 to 6.07	−2.47	−2.88 to −2.07	<0.00001	0.963
	National	62	12.28%	6.00	5.94	5.36 to 6.51	−3.01	−3.62 to −2.40	<0.00001	1.33
Medical expenses	OurPower	186	24.06%	6.00	6.19	5.82 to 6.56	−2.89	−3.32 to −2.47	<0.00001	1.15
	National	61	12.08%	6.00	5.72	5.13 to 6.31	−2.76	−3.39 to −2.13	<0.00001	1.20
Home repairs	OurPower	132	17.08%	5.00	5.42	4.92 to 5.92	−1.71	−2.25 to −1.16	<0.00001	0.62
	National	56	11.09%	5.00	5.20	4.52 to 5.87	−2.14	−2.84 to −1.43	<0.00001	0.897
Rent or mortgage	OurPower	97	12.55%	6.00	6.18	5.68 to 6.67	−2.49	−3.02 to −1.95	<0.00001	0.927
	National	38	7.52%	5.50	5.66	4.85 to 6.47	−2.56	−3.39 to −1.72	<0.00001	1.07
Cleaning products	OurPower	124	16.04%	6.00	5.92	5.48 to 6.36	−2.29	−2.77 to −1.80	<0.00001	0.854
	National	31	6.14%	4.00	4.87	4.19 to 5.55	−1.68	−2.39 to −0.965	0.0000287	0.687
Personal hygiene items	OurPower	96	12.42%	6.00	6.25	5.78 to 6.72	−2.57	−3.08 to −2.05	<0.00001	0.96
	National	26	5.15%	6.00	6.27	5.30 to 7.24	−3.14	−4.13 to −2.14	<0.00001	1.32
Water or other utility bills	OurPower	54	6.99%	6.00	6.04	5.37 to 6.70	−2.18	−2.88 to −1.49	<0.00001	0.794
	National	25	4.95%	6.00	5.76	4.63 to 6.89	−2.59	−3.74 to −1.44	0.0000883	1.08
Education expenses	OurPower	100	12.94%	5.50	5.81	5.28 to 6.34	−2.08	−2.64 to −1.51	<0.00001	0.764
	National	18	3.56%	6.00	6.06	5.05 to 7.06	−2.86	−3.88 to −1.84	0.000012	1.18
Menstrual products	OurPower	78	10.09%	6.00	5.95	5.40 to 6.50	−2.16	−2.75 to −1.57	<0.00001	0.792
	National	17	3.37%	7.00	6.00	4.96 to 7.04	−2.80	−3.86 to −1.74	0.0000311	1.15
Nappies or wipes	OurPower	54	6.99%	6.00	6.30	5.56 to 7.03	−2.46	−3.23 to −1.70	<0.00001	0.9
	National	13	2.57%	7.00	6.46	4.89 to 8.03	−3.25	−4.83 to −1.67	0.0000753†	1.13†
Other essential products or services	OurPower	82	10.61%	6.00	5.93	5.38 to 6.48	−2.15	−2.74 to −1.56	<0.00001	0.789
	National	32	6.34%	7.00	6.34	5.60 to 7.09	−3.25	−4.02 to −2.49	<0.00001	1.39
None of the above	OurPower	357	46.18%	2.00	2.46	2.24 to 2.69	2.95	2.61 to 3.29	<0.00001	1.23
	National	297	58.81%	2.00	2.19	1.98 to 2.40	2.69	2.29 to 3.08	<0.00001	1.28
Do not know or prefer not to answer	National	30	5.94%	4.00	4.17	3.44 to 4.89	−0.927	−1.69 to −0.168	0.0181	0.376

Both OurPower and national surveys presented the largest effect sizes for *Food*, with strong Cohen's d of 1.36 and 1.45, respectively.

#### 3.3.10 Disconnection

Both surveys asked respondents if they had been disconnected for lack of payment of gas or electricity bills in the previous twelve months. The relationship between disconnection and the EHPS was analysed via two-tailed Welch's t-tests (Table 12 below).

**Table 12:** Relationship between disconnection and energy hardship point score (two-tailed Welch's *t*-test, with † being ranked *t*-test).

Disconnections in the last 12 months	Survey	Total (n)	Total (%)	Median EHPS	Average EHPS	CI of average EHPS	Average EHPS difference (unselected - selected)	CI of EHPS difference (unselected - selected)	P-value	Effect size
Yes (self-disconnection)	OurPower	6	0.78%	6.50	6.17	4.02 to 8.31	−2.22	−4.36 to −0.0839	0.0218†	0.860†
	National	3	0.59%	8.00	7.67	3.87 to 11.46	–	–	–	–
Yes (by the provider)	OurPower	53	6.86%	6.00	6.74	6.04 to 7.43	−2.98	−3.70 to −2.26	<0.00001†	1.26†
	National	12	2.38%	6.50	6.75	5.26 to 8.24	−3.54	−5.04 to −2.04	0.000261	1.46
No	OurPower	711	91.98%	3.00	3.75	3.55 to 3.95	2.89	2.19 to 3.60	<0.00001	1.07
	National	486	96.24%	3.00	3.17	2.95 to 3.38	3.41	2.34 to 4.49	<0.00001	1.43
Do not know or prefer not to answer	National	4	0.79%	4.50	5.25	2.24 to 8.26	−1.97	−4.96 to 1.02	0.0342†	0.890†

The sample size for selecting *Yes (self-disconnection)* in the national survey was too small, so no analyses were performed. Selecting *Yes (self-disconnection)* added a point towards the EHPS. For both surveys, selecting *Yes (by the provider)* had the strongest effect sizes (Cohen's d for OurPower being 1.26 and 1.46 for the national survey).

#### 3.3.11 Energy-saving behaviour

Respondents answered if they regularly perform energy-saving behaviours. One-way ANOVA test using Welch's F-test determined the strength of the relationship between this variable and the EHPS. Both surveys presented high P-values (0.983 for OurPower and 0.160 for the national), and low effect sizes (Cohen's f of 0.00205 for OurPower and 0.0869 for the national survey). This means that the relationship was statistically insignificant.

#### 3.3.12 Energy efficiency

Both surveys asked respondents about the presence of energy efficient items and upgrades in their dwellings, including thermal insulation. Two-tailed Welch's t-tests linked the options with the EHPS Some options were not statistically significant. For both surveys, the highest effect sizes were strong (Cohen's d of 0.815 for OurPower and 0.822 for the national one), corresponding to selecting *Windows*.

### 3.4 Dampness

Respondents selected the frequency their home was perceived as damp. One-way ANOVA test using Welch's F-test was performed to analyse the strength between this variable and the EHPS. Both surveys presented P-values<0.00001 and strong effect sizes (Cohen's f of 0.788 for OurPower and 0.776 for the national survey). Selecting *Always* counted as a point towards the EHPS.

### 3.5 Mould

Survey respondents answered if they home had mould. The relationship between mould presence and the EHPS was done via one-way ANOVA test using Welch's F-test. P-values<0.00001 were found for both surveys, in addition to high effect sizes. The Cohen's f for OurPower was 0.596, whereas the national survey's was 0.633. *Always (mould area larger than an A4 sheet)* added a point to the EHPS.

### 3.6 Different statements

Respondents were asked a series of statements about their home and household in the past twelve months, and they selected which ones applied to them. Two-tailed Welch's t-tests were performed to analyse the relationship between each statement and the EHPS (Table 13 below).

**Table 13:** Relationship between different statements and energy hardship point score (two-tailed Welch's *t*-test, with † meaning ranked *t*-test).

Statements	Survey	Total (n)	Total (%)	Median EHPS	Average EHPS	CI of average EHPS	Average EHPS difference (unselected - selected)	CI of EHPS difference (unselected - selected)	P-value	Effect size
In winter, my home gets cold enough that I can see breath indoors	OurPower	204	26.39%	7.00	6.62	6.32 to 6.93	−3.6	−3.96 to −3.24	<0.00001†	1.52†
	National	84	16.63%	7.00	6.45	5.94 to 6.96	−3.79	−4.33 to −3.25	<0.00001	1.86
My household accrued debt on electricity/gas account	OurPower	47	6.08%	7.00	6.68	5.92 to 7.44	−2.88	−3.67 to −2.10	<0.00001	1.06
	National	22	4.36%	7.00	6.68	5.65 to 7.72	−3.54	−4.60 to −2.49	<0.00001	1.49
My household has been unable to pay the electricity/gas bill by the due date >1x	OurPower	104	13.45%	7.00	6.63	6.115 to 7.12	−3.07	−3.60 to −2.55	<0.00001	1.18
	National	35	6.93%	6.00	5.71	4.78 to 6.65	−2.60	−3.56 to −1.64	<0.00001	1.09
In winter, my home gets cold enough that I shiver	OurPower	265	34.28%	6.00	5.76	5.45 to 6.07	−2.72	−3.10 to −2.34	<0.00001	1.09
	National	121	23.96%	5.00	5.44	5.02 to 5.86	−2.82	−3.29 to −2.35	<0.00001	1.30
My household had to make a payment plan or switch to prepay	OurPower	52	6.73%	7.00	7.02	6.30 to 7.73	−3.26	−4.01 to −2.52	<0.00001	1.22
	National	27	5.35%	5.00	4.96	3.95 to 5.97	−1.76	−2.79 to −0.733	0.00153	0.721
My household's primary language is English	OurPower	538	69.60%	3.00	3.66	3.43 to 3.90	1.02	0.597 to 1.45	<0.00001	0.37
	National	413	81.78%	3.00	3.11	2.87 to 3.35	1.02	0.467 to 1.58	0.000384	0.418
My home is in a good shape and does not need any major repairs	OurPower	416	53.82%	2.00	2.36	2.17 to 2.54	3.5	3.19 to 3.82	<0.00001	1.60
	National	277	54.85%	2.00	1.90	1.71 to 2.10	3.08	2.73 to 3.44	<0.00001	1.59
My household has been able to afford a 500 NZD expense without borrowing money	OurPower	276	35.71%	2.00	2.11	1.86 to 2.36	2.90	2.56 to 3.24	<0.00001	1.190
	National	258	51.09%	2.00	2.00	1.79 to 2.22	2.64	2.27 to 3.01	<0.00001	1.26
My household has an electric vehicle that is charged at home	National	11	2.18%	3.00	2.91	1.22 to 4.59	0.395	−1.30 to 2.09	0.637†	0.159†
None of those	National	10	1.98%	4.50	4.60	3.12 to 6.08	−1.33	−2.82 to 0.155	0.0159†	0.632†

Selecting *In winter, my home gets cold enough that I can see breath indoors* and *My household has been unable to pay the energy bill on time* > *1x* counted as one point towards the EHPS each, as well as not selecting *My home is in a good shape and does not need any major repairs* and *My household has been able to afford a 500 NZD expense without borrowing*.

*In winter, my home gets cold enough that I can see breath indoors* had the highest effect size for the national survey (Cohen's d = 1.86). For the OurPower survey, *My home is in a good shape and does not need any major repairs* had the highest effect size (Cohen's d = 1.60).

## 4 Discussion

### 4.1 Most relevant indicators

**Fig. 3:**
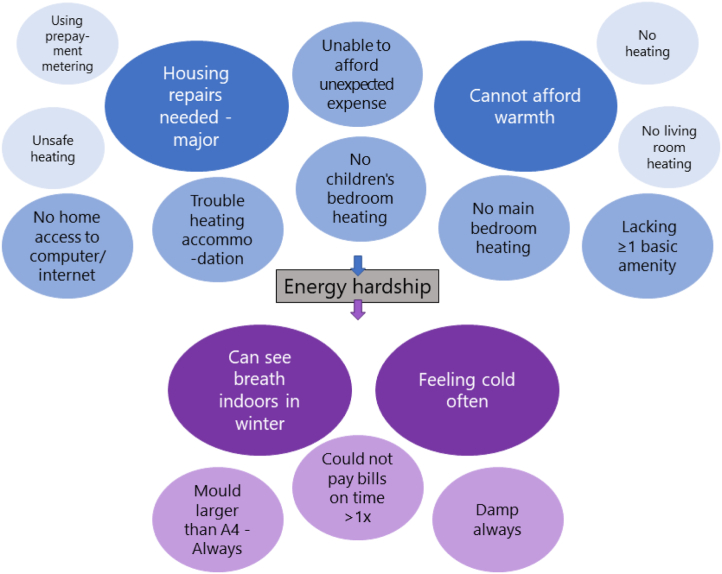
Indicators separated as causes (blue) and consequences (purple) of energy hardship in the OurPower survey (larger sizes with darker shades meaning most relevant ones).

Using Qualtrics, we selected all the indicators and related the scores of both surveys. The results were sorted from most to least relevant indicator. For OurPower, the top indicator was household feeling cold often (Table 14 and Fig. 3 below). As for the national survey, it was major housing repairs needed (Table 15 and Fig. 3 below).

**Table 14:** Strong relationships between indicators and energy hardship point score (OurPower survey).

**P3: Feeling cold often**	P-value	<0.00001	**P6: No home access to computer or internet**	P-value	<0.00001
	Effect size (Pearson's r)	0.645		Effect size (ρ)	0.365
	CI of effect size	0.602 to 0.685		CI of effect size	0.302 to 0.425
	Test used	Correlation		Test used	Ranked correlation
**P22: Housing repairs needed - major**	P-value	<0.00001	**P8: Could not pay bills on time** > **1x**	P-value	<0.00001
	Effect size (Pearson's r)	0.624		Effect size (ρ)	0.356
	CI of effect size	0.579 to 0.665		CI of effect size	0.293 to 0.416
	Test used	Correlation		Test used	Ranked correlation
**P13: Cannot afford warmth**	P-value	<0.00001	**P21: Lacking one or more basic amenity**	P-value	<0.00001
	Effect size (Pearson's r)	0.585		Effect size (ρ)	0.332
	CI of effect size	0.536 to 0.629		CI of effect size	0.268 to 0.393
	Test used	Correlation		Test used	Ranked correlation
**P25: Can see breath indoors in winter**	P-value	<0.00001	**P17: No children's bedroom heating**	P-value	<0.00001
	Effect size (Pearson's r)	0.566		Effect size (Pearson's r)	0.324
	CI of effect size	0.516 to 0.612		CI of effect size	0.260 to 0.386
	Test used	Correlation		Test used	Correlation
**P9: Unable to afford unexpected expense**	P-value	<0.00001	**P23: Mould larger than an A4 - Always**	P-value	<0.00001
	Effect size (Pearson's r)	0.496		Effect size (ρ)	0.315
	CI of effect size	0.441 to 0.548		CI of effect size	0.250 to 0.377
	Test used	Correlation		Test used	Ranked correlation
**P16: No main bedroom heating**	P-value	<0.00001	**P14: Using prepayment metering**	P-value	<0.00001
	Effect size (Pearson's r)	0.441		Effect size (ρ)	0.273
	CI of effect size	0.382 to 0.496		CI of effect size	0.206 to 0.337
	Test used	Correlation		Test used	Ranked correlation
**P24: Damp always**	P-value	<0.00001	**P18: No living room heating**	P-value	<0.00001
	Effect size (ρ)	0.424		Effect size (ρ)	0.229
	CI of effect size	0.364 to 0.480		CI of effect size	0.161 to 0.295
	Test used	Ranked correlation		Test used	Ranked correlation
**P19: Trouble heating accommodation**	P-value	<0.00001	**P15: No heating**	P-value	<0.00001
	Effect size (Pearson's r)	0.396		Effect size (ρ)	0.224
	CI of effect size	0.335 to 0.454		CI of effect size	0.156 to 0.290
	Test used	Correlation		Test used	Ranked correlation
			**P20: Unsafe heating**	P-value	<0.00001
				Effect size (ρ)	0.222
				CI of effect size	0.154 to 0.288
				Test used	Ranked correlation

**Fig. 4:**
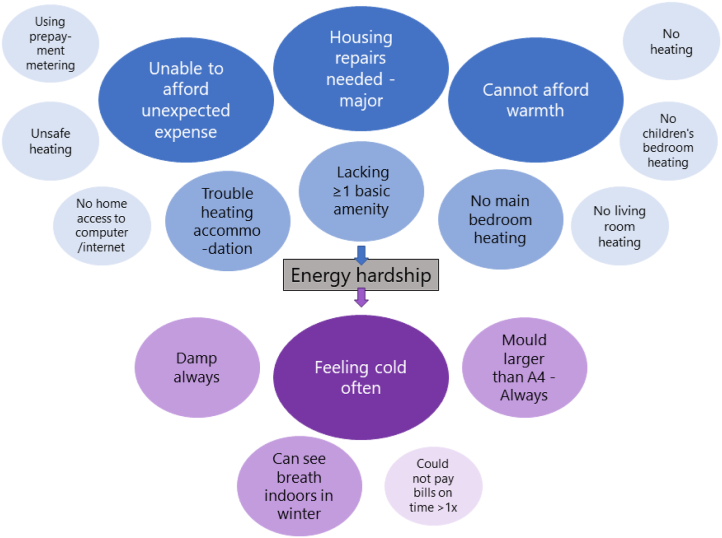
Indicators separated as causes (blue) and consequences (purple) of energy hardship in the national survey (larger sizes with darker shades meaning most relevant ones).

**Table 15:** Strong relationships between indicators and energy hardship point score (national survey).

**P22: Housing repairs needed - major**	P-value	<0.00001	**P23: Mould larger than an A4 - Always**	P-value	<0.00001
	Effect size (Pearson's r)	0.620		Effect size (ρ)	0.328
	CI of effect size	0.563 to 0.671		CI of effect size	0.248 to 0.404
	Test used	Correlation		Test used	Ranked correlation
**P3: Feeling cold often**	P-value	<0.00001	**P21: Lacking one or more basic amenity**	P-value	<0.00001
	Effect size (Pearson's r)	0.614		Effect size (ρ)	0.312
	CI of effect size	0.557 to 0.666		CI of effect size	0.231 to 0.389
	Test used	Correlation		Test used	Ranked correlation
**P13: Cannot afford warmth**	P-value	<0.00001	**P18: No living room heating**	P-value	<0.00001
	Effect size (ρ)	0.569		Effect size ρ)	0.297
	CI of effect size	0.507 to 0.626		CI of effect size	0.215 to 0.375
	Test used	Ranked correlation		Test used	Ranked correlation
**P9: Unable to afford unexpected expense**	P-value	<0.00001	**P6: No home access to computer or internet**	P-value	<0.00001
	Effect size (Pearson's r)	0.533		Effect size (ρ)	0.294
	CI of effect size	0.467 to 0.593		CI of effect size	0.212 to 0.372
	Test used	Correlation		Test used	Ranked correlation
**P25: Can see breath indoors in winter**	P-value	<0.00001	**P15: No heating**	P-value	<0.00001
	Effect size (ρ)	0.497		Effect size (ρ)	0.285
	CI of effect size	0.429 to 0.560		CI of effect size	0.203 to 0.363
	Test used	Ranked correlation		Test used	Ranked correlation
**P19: Trouble heating accommodation**	P-value	<0.00001	**P17: No children's bedroom heating**	P-value	<0.00001
	Effect size (ρ)	0.418		Effect size (ρ)	0.256
	CI of effect size	0.343 to 0.487		CI of effect size	0.173 to 0.336
	Test used	Ranked correlation		Test used	Ranked correlation
**P16: No main bedroom heating**	P-value	<0.00001	**P8: Could not pay bills on time** > **1x**	P-value	<0.00001
	Effect size (Pearson's r)	0.356		Effect size (ρ)	0.235
	CI of effect size	0.277 to 0.430		CI of effect size	0.150 to 0.315
	Test used	Correlation		Test used	Ranked correlation
**P24: Damp always**	P-value	<0.00001	**P14: Using prepayment metering**	P-value	0.0000822
	Effect size (ρ)	0.351		Effect size (ρ)	0.174
	CI of effect size	0.272 to 0.426		CI of effect size	0.0884 to 0.258
	Test used	Ranked correlation		Test used	Ranked correlation
			**P20: Unsafe heating**	P-value	0.0113
				Effect size (ρ)	0.113
				CI of effect size	0.0256 to 0.198
				Test used	Ranked correlation

According to both surveys, using the cause indicators of major housing repairs needed and household's inability to afford warmth, in addition to the consequence indicator of household feeling cold often to save money (Fig. 4 above), can be a simpler way of measuring energy hardship in Kiwi homes. Those three are the most critical indicators that have other implications about the household conditions and can be self-reported – making it easy to get large sample sizes and compare results with other studies. Furthermore, they can guide policies and interventions to focus on reducing the main causes of energy hardship: housing quality and energy unaffordability.

### 4.2 Most relevant non-indicator variables

Using Qualtrics, we selected all the non-indicator variables and related to the EHPS of both surveys. The results were sorted from most to least relevant issues - only those that presented strong effect sizes (Table 16, Table 17 below). For OurPower, the most relevant variable was choosing food expenses over energy bills or vice-versa, and for the national survey it was accumulating debt on the electricity/gas account.

**Table 16:** Most relevant non-indicator variables associated with the energy hardship point score of the OurPower survey.

Variable	P-value	Effect size
Choosing food expenses over energy bills or vice-versa	<0.00001	Cohen's d = 1.36
Choosing medical expenses over energy bills or vice-versa	<0.00001	Cohen's d = 1.15
Being disconnected by the energy provider	<0.00001	Cohen's d = 1.11
Shivering at home during winter due to cold	<0.00001	Cohen's d = 1.09
Accumulating debt on electricity/gas account	<0.00001	Cohen's d = 1.06
Going without fresh fruit or vegetables to save money	<0.00001	Cohen's d = 1.05
Postponing or putting off visits to the doctor to save money	<0.00001	Cohen's d = 0.991
Receiving or borrowing money from family or friends to pay for energy bills	<0.00001	Cohen's d = 0.980
Choosing transportation expenses over energy bills or vice-versa	<0.00001	Cohen's d = 0.963
Choosing other personal hygiene expenses over energy bills or vice-versa	<0.00001	Cohen's d = 0.960
Not earning enough income to match daily necessities	<0.00001	Cohen's f = 0.697
Choosing rent or mortgage over energy bills or vice-versa	<0.00001	Cohen's d = 0.927
Choosing nappy or wipe expenses over energy bills or vice-versa	<0.00001	Cohen's d = 0.900
Choosing clothing expenses over energy bills or vice-versa	<0.00001	Cohen's d = 0.871
Choosing cleaning product expenses over energy bills or vice-versa	<0.00001	Cohen's d = 0.854
Not having insulated windows	<0.00001	Cohen's d = 0.815
Not having energy-efficient lightbulbs	<0.00001	Cohen's d = 0.810
Taking out a loan to pay for energy bills	0.001	Cohen's d = 0.805
Not heating the home office during winter	<0.00001	Cohen's d = 0.803

**Table 17:** Most relevant non-indicator variables associated with the energy hardship point score of the national survey.

Variable	P-value	Effect size
Accumulating debt on the electricity/gas account	<0.00001	Cohen's d = 1.49
Choosing food expenses over energy bills or vice-versa	<0.00001	Cohen's d = 1.45
Choosing other essential product or service expenses over energy bills or vice-versa	<0.00001	Cohen's d = 1.39
Choosing transportation expenses over energy bills or vice-versa	<0.00001	Cohen's d = 1.33
Choosing other personal hygiene expenses over energy bills or vice-versa	<0.00001	Cohen's d = 1.32
Shivering at home during winter due to cold	<0.00001	Cohen's d = 1.30
Being disconnected by the energy provider	<0.00001	Cohen's d = 1.26
Choosing medical expenses over energy bills or vice-versa	<0.00001	Cohen's d = 1.20
Choosing educational expenses over energy bills or vice-versa	0.000012	Cohen's d = 1.18
Choosing menstrual expenses over energy bills or vice-versa	0.0000311	Cohen's d = 1.15
Receiving or borrowing money from family or friends to pay for energy bills	0.0000322	Cohen's d = 1.15
Choosing nappy or wipe expenses over energy bills or vice-versa	0.0000753	Cohen's d = 1.13
Choosing clothing expenses over energy bills or vice-versa	<0.00001	Cohen's d = 1.08
Choosing water or other utility bills over energy bills or vice-versa	0.0000883	Cohen's d = 1.08
Choosing rent or mortgage over energy bills or vice-versa	<0.00001	Cohen's d = 1.07
Going without fresh fruit or vegetables to save money	<0.00001	Cohen's d = 1.07
Choosing home repair expenses over energy bills or vice-versa	<0.00001	Cohen's d = 0.897
Not having insulated windows	<0.00001	Cohen's d = 0.822
Postponing or putting off visits to the doctor to save money	<0.00001	Cohen's d = 0.809
Delaying the replacement or repair of broken or damaged appliances to save money	<0.00001	Cohen's d = 0.801

If a household decides to self-ration to reduce their energy expenses, it can lead to thermal discomfort (such as shivering) and having health conditions in the long term [36] – and those conditions will be translated into higher health expenses. Energy expenses can also be reduced by having insulated windows and energy-efficient lightbulbs, as well as repairing or retrofitting appliances to consume less energy [37]. However, energy efficient upgrades need an initial expense that many households cannot afford or are unable to perform due to their tenancy status [30,38]. Tenants are also more likely to be low-income, creating more financial pressure for them to decide between paying for rent or energy bills.

While having unutilised broken appliances reduces energy consumption, it can increase domestic labour (traditionally mainly performed by women) to substitute their function [39] and also decrease housing quality, potentially leading to health conditions [40,41]. Although we were able to get a reasonable sample size to represent a country with a small population (about 5.1 million people [42]), we are aware that household surveys tend to obscure power relations and other intra-household dynamics, often related to gender and age.

Having broken appliances can also lead to food being spoilt and having to purchase ready-made meals, which are likely to be unhealthy options and to increase food costs for households [43]. The health is also compromised if the household opts for consuming the spoilt food. Decreasing food expenses (especially when it comes to fruits and vegetables) due to financial restraints can cause health conditions and food insecurity [44,45].

While transportation fuel consumption is not considered as part of the definition of energy hardship in Aotearoa [15,46], transportation expenses can also limit the money available to pay for energy bills, which affects energy hardship and increase existing inequalities relating to right to the city [47,48]. Due to the pandemic, more people started working from home on a temporary or permanent basis [49]. Though it reduces commute expenses, using a home office requires appropriate temperature regulation and ventilation to avoid health conditions [50]. Other than working from home, online learning rather than going to campus has also become more frequent [51]. Educational expenses also limit the money available for energy needs However, being educated can improve the chances of earning higher income in the future.

If purchasing menstrual products, nappies, or wipes are not prioritised, more domestic labour will be needed to improvise solutions, and health conditions can be developed from their inappropriate maintenance [52,53]. Limiting the use of personal hygiene products, cleaning products, utilities (e.g. water), and other vital expenses also lead to health conditions. While saving on clothing can help during times of limited finances, having proper clothes to regulate body temperature is essential for good health, especially with limited heating [54].

Limiting doctor visits for financial reasons can result in worsening health conditions. Those can also increase the household energy needs, such as increased need for heating and cooling or electricity-powered equipment for dependent users [[55], [56], [57]]. Low funds leading can make households accumulate debt in their energy bills and eventually get disconnected by their providers. Both situations result in extra fees, making it even more difficult for poor households [58]. Having family and friends that can give or lend money to pay for energy bills reduces the pressure of that expense and reduces the chances of having debt or being disconnected. Some households may take out a loan to pay for their energy bills during times of financial stress, but eventually they will have to pay off that expense plus interest.

Not having enough money is connected to all issues, making households choose between multiple necessities. The main consequence of the deprivations is the development of health conditions, and those result in health expenses and possible increase in energy needs. More energy needs mean more energy expenses, and without enough income or loved ones helping pay for those expenses, households are vulnerable to accumulating debt in their energy accounts, being disconnected by their providers, or obtaining loans - all leading to less money over time.

While suggesting more available income for households is not a specific nor a realistic energy policy for the moment, energy hardship will not be eradicated while households are still struggling to make ends meet. Reducing energy expenses by continuing to develop efforts to improve energy efficiency can be helpful, especially by insulating windows and providing LED lightbulbs. We encourage the expansions of programmes such as Warmer Kiwi Homes [59], but beyond assistance for homeowners only, as tenants are often the most affected by energy hardship.

Providing energy tariff discounts or other financial help for vulnerable households is encouraged, as well as any combination of assistance combining energy and other issues (e.g. targeting food insecure households for energy benefits). Furthermore, subsidies for appliance repairs and retrofits are useful for minimising energy hardship while also prolonging the lifespan of equipment, reducing waste, and decreasing the need for more raw material extraction [60]. Less energy consumption not only leads to lower energy bills, but also lower greenhouse gas emissions.

Energy hardship is a complex condition that is connected to climate change, health issues, food insecurity, gender issues, and many more. Investing resources on the major causes of energy hardship can greatly increase the wellbeing of New Zealanders and result in savings for the households and the government. Our findings suggest that the seventeen MBIE indicators used on the two surveys we conducted could be simplified as three to easily identifiable energy hardship indicators that are reinforcing: living in a poor quality (housing repairs needed – major) and paying high energy prices causes bills to be too high, so one cannot afford heating it (cannot afford warmth), and they end up self-rationing energy and being cold (feeling cold often).

Additionally, we suggest adding one indicator associated with food insecurity, such as the one we used - *choosing food expenses over energy bills or vice-versa* (cause – but can also be a consequence), and another relating to energy debt - *accumulating debt on the electricity/gas account* (consequence). All of those variables have been thoroughly discussed in international literature and even in New Zealand [20,44,58,61], and this study highlights that the *heat or eat dilemma* and energy debt issue need to have specific policies and should be considered as additional indicators.

While ethnicity, disability, tenancy status, and education level were related to energy hardship to some extent, those were not major factors. Interestingly, living in the South Island was not even statistically significant in our study, which could be due to the fact we did not use indicators such as ratio of energy expenses over income, which has been traditionally used in New Zealand research [13,62]. However, we are interested in seeing future analyses of all twenty-six MBIE indicators in the whole country for better comparison of those specific variables.

Our findings also show that, overall, OurPower customers that responded to the survey are in more energy hardship and general deprivation than respondents of the nationally representative survey (even if compared to those in the Waikato region only). This is most likely because OurPower is a social retailer, catering to low-income households by offering simple electricity plans with low prices, and they do not refuse new customers that have poor credit [25,63]. At the time this manuscript was being written, the New Zealand government was encouraging the expansion of social retailers to minimise energy hardship, and seeking out public feedback on the strategies regarding how that should be carried out [63,64].

We hope our study can be translated into efforts being put into the most critical needs of Aotearoa while also inspiring people in other countries to do the same. We encourage researchers worldwide to associate specific critical material needs to fuel and energy poverty to find potential causes and consequences of the issues, as shown by our study.

Furthermore, respondents of both surveys were asked to respond to a follow-up survey in late 2022 to give updates on their condition and feedback on the survey rewards and other energy hardship interventions. Their responses will be used to evaluate those actions and analyse the best practices and propose supplementary interventions for energy hardship eradication in New Zealand.

## 5 Conclusion

We identified that the main causes of energy hardship in Aotearoa New Zealand in 2022 were the household's inability to afford sufficient warmth (indicator), major housing repairs needed (indicator), and the household having to choose between paying for food or energy bills. The household feeling cold often due to self-rationing (indicator) and accumulating energy debt were major consequences. We suggest for energy hardship policies to be aimed at increasing energy affordability for vulnerable households, improving housing quality and energy efficiency, as well as integrating efforts with other social programmes in the country. Reducing energy hardship will ameliorate the health and wellbeing of Kiwi households and minimise their financial stress.

## Ethical statement

This study was granted Ethics Approval for Research by the Waikato Management School Human Research Ethics Committee on May 2, 2022 (Ethical Application WMS 22/19).

## Author contribution statement

Luiza Brabo-Catala: Conceived and designed the experiments; Performed the experiments; Analysed and interpreted the data; Contributed reagents, materials, analysis tools or data; Wrote the paper. Anca Cernic: Analysed and interpreted the data; Contributed reagents, materials, analysis tools or data; Wrote the paper. Anca Cernic: Conceived and designed the experiments; Analysed and interpreted the data; Wrote the paper. Barry Barton: Analysed and interpreted the data; Wrote the paper.

## Data availability statement

Data will be made available on request.

## Declaration of competing interest

The authors declare the following financial interests/personal relationships which may be considered as potential competing interests:Note: The authors report that the organisations mentioned in the manuscript (OurPower, Habitat for Humanity Northern Region, Northpower, and Orion provided survey rewards for the respondents.
