# Supplementary material for: The heat goes on: Simplifying the identification of energy hardship
Source: Heliyon. 2023 Aug 11;9(8):e19087. doi: 10.1016/j.heliyon.2023.e19087 (PMC10450965; doi:10.1016/j.heliyon.2023.e19087)
Supplement: Multimedia component 1 [file mmc1.docx]

# Appendix A: Questions

Questions relating to terms of agreement and rewards were not included. Possible answers were shown in the results section.

## OurPower survey questions

- What is the postcode of your residence?
- In which region does your household live in? [if previous was empty]
- How many people reside in your household, including yourself? Write the number of people per age group ("0" if none).
- What are the ethnicities of the people who live in your household? Select all that apply.
- What is the highest education level achieved by a household member?
- How would you describe the general health of people in your household (including yourself)?
- In the last two weeks, how often have you:

- felt cheerful and in good spirits?

- felt calm and relaxed?

- felt active and vigorous?

- woken up feeling fresh and rested?

- felt that your daily life has been filled with things that interest you?

- Are there any household members with a disability or an impairing chronic illness? Select all that apply.
- How many household members stay at home full-time (e.g. working from home, retired, unemployed, not going to school)? Write the number of people staying at home full-time per age group ("0" if none).
- What is the total annual gross income of your household in NZD? Write the number (e.g. 34500.92). Click here to use an income calculator, if needed.
- Which group best represents the total annual gross income of your household? Click here to use an income calculator, if needed. [if previous was empty]
- How well does your household’s total income meet your everyday needs, for such things as accommodation, food, clothing, and other necessities?
- In the last 12 months, have you done any of the following things to keep costs down? Select all that apply.
- What is your household's accommodation situation? Select all that apply.
- Select the fuels used by your household during winter months (June, July, and August only). Consider fuels used to run appliances, indoor heating/cooling, water heating, and cooking. Disregard transportation fuel.
- How much does your household spend on energy during winter months (June, July, and August only)? Consider fuels used to run appliances, indoor heating/cooling, water heating, and cooking. Disregard transportation fuel. Add the average monthly expense per selected group.
- Select the fuels used by your household during non-winter months (all months except for June, July, and August). Consider fuels used to run appliances, indoor heating/cooling, water heating, and cooking. Disregard transportation fuel.
- How much does your household spend on energy during non-winter months (all months except for June, July, and August)? Consider fuels used to run appliances, indoor heating/cooling, water heating, and cooking. Disregard transportation fuel. Add the average monthly expense per selected group.
- Which of the following types of heating, if any, do you have in your home? Select all that apply.
- Is your home always adequately warm during winter? Select all that apply.
- Select all types of rooms you have in your home.
- Select all the rooms you heat in your home during winter.
- Did you feel your dwelling was too hot during last summer (2021/2022)?
- In the last 12 months, has your household ever had to choose between paying energy bills or any of the following? Select all that apply.
- Does your household regularly perform energy-saving behaviours such as drying clothes naturally, taking shorter showers, and turning off the lights?
- In the last 12 months, has your household been disconnected (or self-disconnected if you prepay) for late or non-payment of electricity or gas bill? Select all that apply.
- During the last 12 months, has your household received any financial assistance to help pay for energy services? Select all that apply.
- During the past 12 months, how often have any of your household members used medical, assistance, or mobility equipment at home which required energy consumption (e.g. oxygen concentrators and motorised wheelchairs)?
- Does your home have any thermal insulation/energy efficiency upgrades in any of the following? Select all that apply.
- Approximately, in what decade was your home built?
- Does your home have any of the following amenities? Select all that apply.
- Would you say your home is always damp, sometimes damp, or not damp at all (e.g. damp floors, foundation, walls, or leaking roof)?
- Does any part of your home have mould growing on it (e.g. walls, ceiling, window frames, curtains, or blinds)?
- Which of the following statements apply to your household from the last 12 months until now? Select all that apply.

## National survey questions

- In which region do you live in?
- How many people reside in your household, including yourself, in each of the groups below? Please type in a number in each text box ("0" if none).
- What are the ethnicities of the people who live in your household (including yourself)? Select all that apply.
- What is the highest education level achieved by a member of your household?
- How would you describe the general health of people in your household (including yourself)?
- In the last two weeks, how often have you:

- felt cheerful and in good spirits?

- felt calm and relaxed?

- felt active and vigorous?

- woken up feeling fresh and rested?

- felt that your daily life has been filled with things that interest you?

- Are there any household members with a disability or an impairing chronic illness? Select all that apply.
- How many household members stay at home full-time (e.g. working from home, retired, unemployed, not going to school)? Write the number of people staying at home full-time for each group. If no members stay at home full time please type in "0".
- What is the total annual gross income (before tax and other deductions) of your household in NZD? Write the number of NZD below (e.g. 34500.92). Click here to use an income calculator, if needed.
- Which group best represents the total annual gross income (before tax and other deductions) of your household? Click here to use an income calculator, if needed. [if previous was empty]
- How well does your household’s total income meet your everyday needs, for such things as accommodation, food, clothing, and other necessities?
- In the last 12 months, have you done any of the following things to keep costs down? Select all that apply.
- What is your household's accommodation situation?
- Select the fuels used by your household during winter months (June, July, and August only). Include fuels used to run appliances, indoor heating/cooling, water heating, and cooking. Exclude transportation fuel.
- How much does your household spend on energy during winter months (June, July, and August only)? Include fuels used to run appliances, indoor heating/cooling, water heating, and cooking. Exclude transportation fuel. Add the average monthly expense for each group shown below.
- Select the fuels used by your household during non-winter months (all months except for June, July, and August). Include fuels used to run appliances, indoor heating/cooling, water heating, and cooking. Exclude transportation fuel.
- How much does your household spend on energy during non-winter months (all months except for June, July, and August)? Include fuels used to run appliances, indoor heating/cooling, water heating, and cooking. Exclude transportation fuel. Add the average monthly expense for each group shown below.
- Which of the following types of heating, if any, do you have in your home? Select all that apply.
- Is your home always adequately warm during winter? Select all that apply.
- Select all types of rooms you have in your home.
- Select all the rooms you heat in your home during winter.
- Did you feel your dwelling was too hot during last summer (2021/2022)?
- In the last 12 months, has your household ever had to choose between paying energy bills or any of the following? Select all that apply.
- Does your household regularly perform energy-saving behaviours such as drying clothes naturally, taking shorter showers, and turning off the lights?
- In the last 12 months, has your household been disconnected from electricity or gas (or self-disconnected if you prepay) for late or non-payment of bill? Select all that apply.
- During the last 12 months, has your household received any financial assistance to help pay for energy services? Select all that apply.
- During the past 12 months, how often have any of your household members used medical, assistance, or mobility equipment at home which required energy consumption (e.g. oxygen concentrators and motorised wheelchairs)?
- Does your home have any thermal insulation/energy efficiency upgrades in any of the following? Select all that apply.
- Thinking of the home you live in, approximately in what decade was it built?
- Does your home have any of the following amenities? Select all that apply.
- Would you say your home is always damp, sometimes damp, or not damp at all (e.g. damp floors, foundation, walls, or leaking roof)?
- Does any part of your home have mould growing on it (e.g. walls, ceiling, window frames, curtains, or blinds)?
- Which of the following statements apply to your household considering the last 12 months until now? Select all that apply.

# Appendix B: MBIE indicators

| **Indicator** | **Survey** | **Total (n)** | **Total (%)** |  | **Indicator** | **Survey** | **Total (n)** | **Total (%)** |
| --- | --- | --- | --- | --- | --- | --- | --- | --- |
| Unable to afford unexpected expense without borrowing* | OurPower | 497 | 64.29% |  | Could not pay electricity, gas, rates, or water bills on time (more than once)** | OurPower | 104 | 13.45% |
|  | National | 247 | 48.91% |  |  | National | 35 | 6.93% |
| Housing repairs needed - major | OurPower | 357 | 46.18% |  | No home access to computer or internet | OurPower | 100 | 12.94% |
|  | National | 282 | 55.84% |  |  | National | 34 | 6.73% |
| Not heating own bedroom in winter | OurPower | 370 | 47.87% |  | Damp always | OurPower | 81 | 10.48% |
|  | National | 230 | 45.54% |  |  | National | 29 | 5.74% |
| Put up with feeling cold to keep costs down a lot | OurPower | 295 | 38.16% |  | Mould larger than an A4 - Always | OurPower | 64 | 8.28% |
|  | National | 153 | 30.30% |  |  | National | 38 | 7.52% |
| Cannot afford to keep the dwelling adequately warm | OurPower | 267 | 34.54% |  | Not heating main living room in winter | OurPower | 44 | 5.69% |
|  | National | 126 | 24.95% |  |  | National | 42 | 8.32% |
| Trouble heating accommodation and/or keeping it warm in winter | OurPower | 240 | 31.05% |  | Using (or having used) prepayment metering*** | OurPower | 55 | 7.12% |
|  | National | 116 | 22.97% |  |  | National | 29 | 5.74% |
| Not heating children's bedroom in winter | OurPower | 225 | 29.11% |  | Use of unsafe substitute heating methods (portable gas heater) | OurPower | 43 | 5.56% |
|  | National | 120 | 23.76% |  |  | National | 33 | 6.53% |
| Can see breath indoors in winter | OurPower | 204 | 26.39% |  | No heating type used | OurPower | 27 | 3.49% |
|  | National | 84 | 16.63% |  |  | National | 17 | 3.37% |
| Lacking one or more basic amenity | OurPower | 99 | 12.81% |  |  |  |  |  |
|  | National | 70 | 13.86% |  |  |  |  |  |

*500 NZD expense.

**We specifically asked for energy bills (electricity and gas).

***Prepayment or energy plan.
